# Splice-site mutation causing partial retention of intron in the *FLCN* gene in Birt-Hogg-Dubé syndrome: a case report

**DOI:** 10.1186/s12920-018-0359-5

**Published:** 2018-05-02

**Authors:** Mitsuko Furuya, Hironori Kobayashi, Masaya Baba, Takaaki Ito, Reiko Tanaka, Yukio Nakatani

**Affiliations:** 1 Department of Molecular Pathology, Yokohama City University Graduate School of Medicine, 3-9 Fukuura, Kanazawa-ku, Yokohama, 236-0004 Japan; 2 Department of Thoracic Surgery, Kumamoto Saishunso National Hospital, Kumamoto, Japan; 3 International Research Center for Medical Sciences, Kumamoto University, Kumamoto, Japan; 4 Department of Diagnostic Pathology, Kumamoto Saishunso National Hospital, Kumamoto, Japan; 5 Medical Mycology Research Center, Chiba University, Chiba, Japan; 6 Department of Diagnostic Pathology, Chiba University Graduate School of Medicine, Chiba, Japan

**Keywords:** Birt-Hogg-Dubé syndrome (BHD), Splice-site mutation, Folliculin (FLCN)

## Abstract

**Background:**

Birt-Hogg-Dubé syndrome (BHD) is an autosomal dominant disorder caused by germline mutations in the folliculin gene (*FLCN*). Nearly 150 pathogenic mutations have been identified in *FLCN*. The most frequent pattern is a frameshift mutation within a coding exon. In addition, splice-site mutations have been reported, and previous studies have confirmed exon skipping in several cases. However, it is poorly understood whether there are any splice-site mutations that cause translation of intron regions in *FLCN*.

**Case presentation:**

A 59-year-old Japanese patient with multiple pulmonary cysts and pneumothorax was hospitalized due to dyspnea. BHD was suspected and genetic testing was performed. The patient exhibited the splice-site mutation of *FLCN* in the 5′ end of intron 9 (c.1062 + 1G > A). Total mRNA was extracted from pulmonary cysts, and RT-PCR assessment and sequence analyses were done. Two distinct bands were generated; one was wild-type and the other was a larger-sized mutant. Sequence analysis of the latter transcript revealed the insertion of 130 base pairs of intron 9 from the beginning of the splice-site between exons 9 and 10.

**Conclusion:**

To our knowledge, this is the first report of distinct intron insertion using a BHD patient’s diseased tissue-derived mRNA. The present case suggests that a splice-site mutation can lead to exon skipping as well as intron reading mRNA. The splicing process may be dependent in part on whether the donor or acceptor site is affected.

**Electronic supplementary material:**

The online version of this article (10.1186/s12920-018-0359-5) contains supplementary material, which is available to authorized users.

## Background

Birt-Hogg-Dubé syndrome (BHD), also called Hornstein-Knickenberg syndrome, is an inherited disorder characterized by skin fibrofolliculomas, multiple pulmonary cysts and kidney cancers [1, 2]. The gene responsible for BHD, folliculin (*FLCN*), is located at 17p11.2. [3], and its protein product, FLCN, cooperatively interacts with its partners folliculin-interacting proteins 1 (FNIP1) and FNIP2, playing important roles in organogenesis and tissue homeostasis [4–8]. The FLCN complex binds with AMPK and regulates mammalian target of rapamycin (mTOR) [4–8]. The principal role of FLCN in human diseases is tumor suppression. Around 20–30% of affected family members are reported to develop renal cell carcinomas (RCCs) [2]. Rats carrying a mutant *Flcn* and heterozygous knockout mice developed renal tumors [9–12]. Further studies using organ-specific *Flcn* knockout mice demonstrated characteristic disorders such as polycystic kidney disease, cardiac hypertrophy and alveolar enlargement, indicating that FLCN function is involved in a wide variety of human disorders [13–16].

There are nearly 150 known mutation patterns of *FLCN* [17, 18]. The most frequent type is a frameshift within an exon region. Less frequent types include nonsense, in-frame deletion, missense, and splice-site mutations. There are also intragenic deletions and duplications [19, 20]. According to the literature, splice-site mutations have been reported between introns 4–13, and there are at least 19 different splice-site mutation patterns [17, 20]. Some of these splice-site mutations are predicted to cause exon skipping [21, 22]. Several cases possess truncated *FLCN* mRNAs with exon skipping as determined by RT-PCR [23–25]. Recently, a study of splice-site mutation cases involving the 5′-end of intron 9 (c.1062 + 2 T > G) demonstrated 2 mutants; composed of a short band lacking exon 9 and a large band reading an additional 130 base pairs (bp) of intron 9 [26]. The large band was much fainter than the short one, and interpreted as a cryptic splice-site [26]. It is not completely understood whether some splice-site mutations aberrantly translate intron regions, forming unconventional transcriptional products in the affected organs of BHD patients. In the current study, we described a case of BHD in which *FLCN* mRNAs had splicing aberrations due to translating a part of an intron.

## Case presentation

### Clinical course

**Fig. 1:**
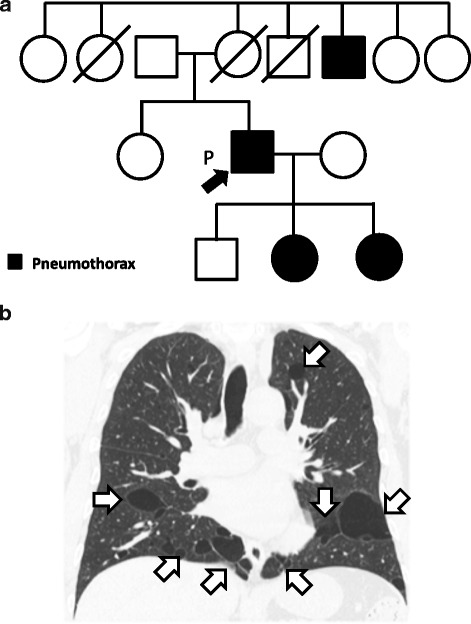
Family tree and radiological findings**. (a)** The patient is indicated by an arrow. Four members including the patient had episodes of pneumothorax, indicated by black. **(b)** Computed tomography shows multiple pulmonary cysts (indicated by arrows)

**Fig. 2:**
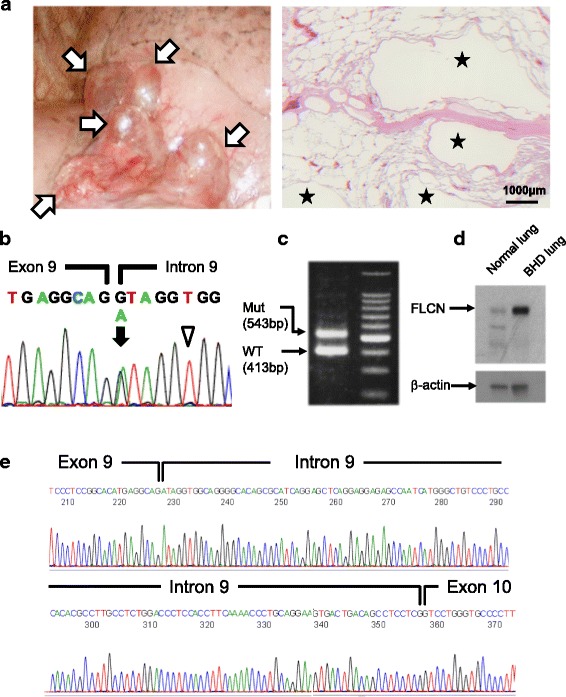
Lung histology and FLCN analysis. **(a)** Left: The thoracoscopy revealed multiple subpleural cysts (arrows). Right: Histology of the resected lung. Pulmonary cysts preferentially develop along an interlobular septum, and they are partially incorporated within alveoli. Neither fibrosis nor active inflammation was observed. Stars indicate cyst lumens. **(b)** Direct sequencing of the *FLCN* gene. The 5′-end of intron 9 was heterozygously mutated to adenine (arrow; c.1062 + 1G > A). A homozygous SNP was also detected (arrowhead; c.1062 + 6C > T). **(c)** RT-PCR of *FLCN* mRNA between exons 8–11. Two products were detected; a wild type (WT) and a mutant (Mut). **(d)** Western blotting of FLCN in the patient’s lung. Normal lung was used for comparison. The FLCN bands of 64 kDa were detected in both lanes. No additional band was observed in the BHD patient’s lung. **(e)** Sequence analysis of mutant RT-PCR product. Intron 9 (130 bp) retention was detected between exons 9–10

A 59-year-old Japanese man was admitted to Kumamoto Saishunso National Hospital for treatment of spontaneous pneumothorax. Now an ex-smoker, he had previously smoked 10–12 cigarettes per day for 25 years. He had not previously experienced a pneumothorax. He did not have fibrous papules on his face or neck. His medical history was unremarkable except for a benign colon polyp at the age of 58. His two daughters and an uncle on the maternal side had episodes of pneumothoraces (Fig. 1a). His mother had died of cervical cancer, and had had no episode of pneumothorax. Computed tomography showed multiple pulmonary cysts (Fig. 1b). Renal tumors were not detectable. He underwent pulmonary wedge resection via video assisted thoracoscopic surgery (VATS). Intrathoracic observation revealed transparent cysts 3–30 mm in diameter distributed in the pleura (Fig. 2a, left). After VATS, the patient has recovered without complication, and has been receiving periodic medical check-ups. From the family history and radiological and clinical findings, the patient was suspected of BHD.

### Pathological finding

The resected lung tissue contained several cystic lesions. Microscopically, these cysts were incorporated into peripheral alveolar tissue in one region and interstitial tissue or visceral pleura in another area (Fig. 2a, right). Most of the cysts developed in contact with bronchovascular bundles and/or interlobular septa. Flattened pneumocytes lined the inner surface of the cysts, and the lining cells were frequently exfoliated from the wall. The cyst walls showed neither fibrous reaction nor active inflammation, which was distinctively different from the histology of emphysematous bullae. Other cystic lung disorders such as lymphangioleiomyomatosis and chronic obstructive pulmonary disease were also ruled out. The characteristic histological features were consistent with BHD-associated pulmonary cysts [27, 28].

### Mutation and expression analysis

Genetic counseling was performed, and informed consent was obtained from the patient for *FLCN* genetic testing and related molecular studies after approval of the Institutional Review Board (IRB) of Yokohama City University. Genetic analysis of *FLCN* identified a single nucleotide mutation at the 5′-end of intron 9 (c.1062 + 1G > A) (Fig. 2b). The mutation had been reported as pathogenic in a previous study [21]. In addition, a single nucleotide polymorphism (SNP) was detected in the vicinity of the splice-site mutation (c.1062 + 6C > T). The patient was finally diagnosed with BHD.

Previous studies have identified several sites of exon skipping in *FLCN* mRNAs in patients with BHD [23–25]. The mutation in intron 9 (c.1062 + 1G > A) was predicted to cause aberrant *FLCN* mRNA, but had not been investigated. We therefore analyzed the patient’s *FLCN* at mRNA and protein levels. Using primers covering exons 8–11 with RT-PCR, two products were clearly detected, i.e., a wild-type band (413 bp) and a larger-sized band between 500 and 600 bp (Fig. 2c). No candidate band for exon skipping was observed. In Western blotting, predicted size FLCN bands were detected in the BHD lung as well as in a normal lung. No other specific band was detected in the patient’s lung (Fig. 2d). We further performed sequence analysis of the RT-PCR products. A wild-type band was confirmed to be composed of exons 9–10. On the other hand, the larger-sized band of the mutant allele was found between exons 9–10, extending from the beginning of intron 9 for 130 bp (Fig. 2e). The size of the mutant mRNA was determined to be 543 bp. The complete size of intron 9 is 1836 bp; however, no larger band was detectable. Moreover, we did not observe other candidates predicting exon skipping. Reanalysis with RT-PCR using different primers designed between exons 8–13 showed again an identical 130 bp intron inserting between exons 9–10 (data not shown). The amino acid sequence predicted by the mutant mRNA caused a frameshift of exon 10, resulting in premature termination after reading 77 amino acids from the 5′-end of intron 9 (Additional file 1 Figure S1).

## Discussion and conclusions

Splice-site mutations are expected to affect mRNA translation by either skipping the adjacent exon or misreading the affected intron. Splice-sites are composed of acceptor sites (the 3′-end of the intron) and donor sites (the 5′-end of the intron). In BHD families, there are more acceptor site than donor site mutations [17]. All of the cases in which exon skipping was confirmed were acceptor site mutations, such as c.397-1G > C, c.1063-2A > G and c.1177-2A > G [23–25]. With regard to donor site mutations, only one study has been reported thus far [26]. Rossing et al. used a mini-gene splicing assay to demonstrate the presence of 2 simultaneous bands in the case of c.1062 + 2 T > G; i.e., an additional 130 bp and an exon skipping [26]. The former sequence was interpreted as a misreading of intron 9 using a cryptic splice-site. Although the mutation of the present case was located in the other nucleotide (c.1062 + 1G > A), both mutations identically read the first 130 bp of intron 9 and connected with exon 10. At the break point of intron 9, retention stopped before a guanine-thymine (GT) sequence (data not shown). It is plausible that the GT sequence just after the break point could have been misunderstood as a donor site. We could not detect exon skipping in the present study, which might owe in part to experimental design. We investigated mRNA expression using the patient’s lung that contained cystic lesions, whereas Rossing et al. used mRNA from transfected COS-7 cells.

A partial intron retention (130 bp) and the following 104 bp of exon 10, which ended at a stop codon, predicted the translation of an additional 77 amino acids between intron 9 (43 amino acids) and exon 10 (34 amino acids). In the latter amino acids involving exon 10, a frameshift occurred due to a guanine left behind the 43 amino acids. We could not detect candidate bands for the mutant protein in Western blotting analysis. Similar results were obtained in our previous study of another patient’s lung with a splice-site mutation in the acceptor site [24]. Although the possible presence of FLCN variants that are undetectable by currently available antibodies cannot be excluded, these pathogenic mRNAs might be degraded through the nonsense-mediated mRNA decay system. Significant expression of normal-sized FLCN in patients’ lungs indicates that the majority of normal-looking pneumocytes preserve FLCN at the protein level in human BHD lungs. On the other hand, FLCN protein is reduced or severely suppressed in BHD-associated RCCs [29]. Since the mice with *Flcn*-depleted type II pneumocytes resulted in alveolar enlargement [15], undetectable levels of FLCN suppression in a limited number of pneumocytes might also contribute to cyst growth. The characteristic localization of cysts in the vicinity of interlobular septa/bronchovascular bundles and visceral pleura, as well as development of elongated vascular network in subpleural cysts [28], suggest that the cysts may originate from specific areas that would slowly lead to cystic growth of the alveolar epithelium with partial incorporation into stroma. A recent in vitro study of pulmonary fibroblasts from BHD patients demonstrated impaired migration and matrix production abilities, suggesting the roles of stroma at shear stress-prone regions of the lung [30]. In this respect, we observed a histopathological analogy between BHD-associated pulmonary cysts and fibrofolliculomas of the skin, the latter also showing cord-like growth of follicular epithelium in association with the surrounding specialized mesenchyme.

**Fig. 3:**
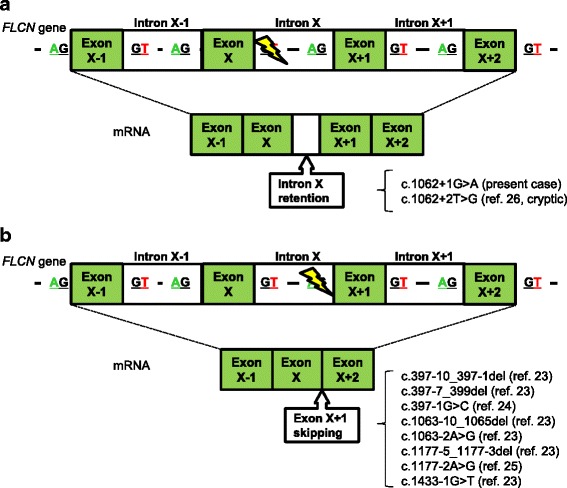
mRNA processing of *FLCN* with splice-site mutations Green boxes are exons and white boxes are introns. Each intron has guanine and thymidine (GT) at the 5′-end and adenine and guanine (AG) at the 3′-end. A yellow notch indicates a mutated region of intron X. **(a)** Two patterns of 5′-end mutation, i.e., the present case and ref. [26], demonstrated intron retention. The latter noted the mutant cryptic. **(b)** Eight patterns of 3′-end mutation (refs. [23–25]) demonstrated exon skipping

The results of the present case and previous studies that demonstrate intron retention and exon skipping are summarized in Fig. 3. We hypothesize that mutations in donor/acceptor sites may play critical roles for determining mRNA processing. The possibilities of aberrant splicing should be considered including condition-dependent processing and cryptic splicing. Further study is needed to determine whether splice-site mutations in other donor sites also cause partial/complete intron retention or produce exon-skipped mRNA in a condition-dependent manner. Better understanding of mRNA processing from mutant *FLCN* will contribute to medical care of BHD patients.

## Additional file

Additional file 1: **Figure S1.** Amino acid sequence predicted by intron retention. Colored nucleotides are exons 9 and 10, and gray nucleotides starting from the mutated adenine (A, indicated by an arrow) are intron insertions. The predicted amino acid sequence is noted below codons in bold. A 130 bp intron retention leads to a frameshift from the beginning of exon 10, which results in premature termination (indicated by a rectangle). (PPTX 43 kb)

## Abbreviations

BHD: Birt-Hogg-Dubé syndrome
*FLCN*: *Folliculin*
VATS: Video assisted thoracoscopic surgery
