# Supplementary material for: Splice-site mutation causing partial retention of intron in the FLCN gene in Birt-Hogg-Dubé syndrome: a case report
Source: BMC Med Genomics. 2018 May 2;11:42. doi: 10.1186/s12920-018-0359-5 (PMC5930857; doi:10.1186/s12920-018-0359-5)
Supplement: Supplementary file 1 — Figure S1. Amino acid sequence predicted by intron retention. Colored nucleotides are exons 9 and 10, and gray nucleotides starting from the mutated adenine (A, indicated by an arrow) are intron insertions. The predicted amino acid sequence is noted below codons in bold. A 130 bp intron retention leads to a frameshift from the beginning of exon 10, which results in premature termination (indicated by a rectangle). (PPTX 43 kb) [file 12920_2018_359_MOESM1_ESM.pptx]

## Slide 1
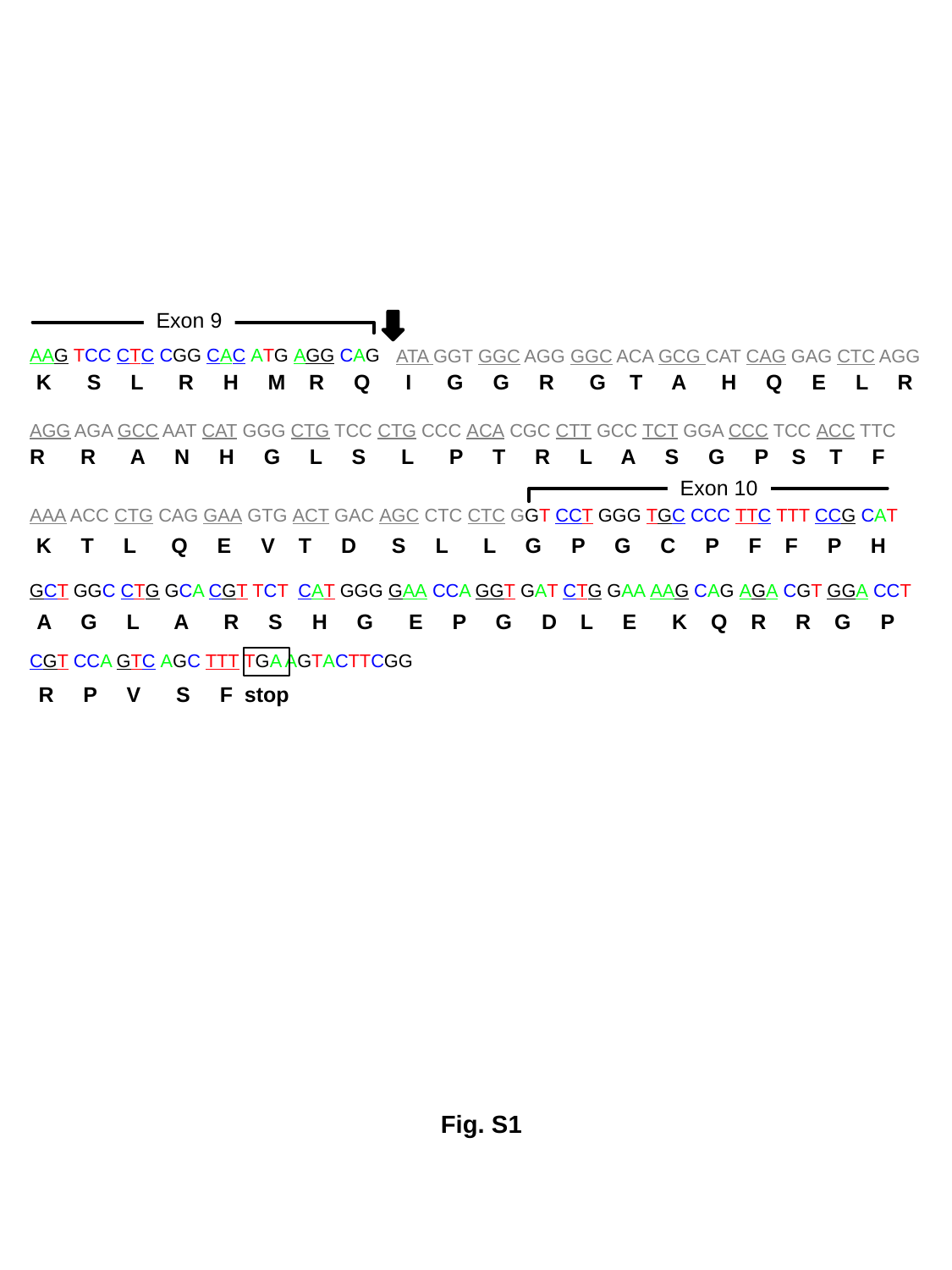

Exon 9
AAG TCC CTC CGG CAC ATG AGG CAG
ATA GGT GGC AGG GGC ACA GCG CAT CAG GAG CTC AGG
K S L R H M R Q I G G R G T A H Q E L R
AGG AGA GCC AAT CAT GGG CTG TCC CTG CCC ACA CGC CTT GCC TCT GGA CCC TCC ACC TTC
R R A N H G L S L P T R L A S G P S T F
Exon 10
AAA ACC CTG CAG GAA GTG ACT GAC AGC CTC CTC GGT CCT GGG TGC CCC TTC TTT CCG CAT
K T L Q E V T D S L L G P G C P F F P H
GCT GGC CTG GCA CGT TCT CAT GGG GAA CCA GGT GAT CTG GAA AAG CAG AGA CGT GGA CCT
A G L A R S H G E P G D L E K Q R R G P
CGT CCA GTC AGC TTT TGA AGTACTTCGG
R P V S F stop
Fig. S1
